# Sanitary Legislation

**Published:** 1858-07

**Authors:** 


### SANITARY LEGISLATION.

*Bills.*—A Bill to Amend the Public Health Act, 1848, and to make further Provision for the Local Government of Towns and Populous Districts, has been prepared by Mr. Adderley and Mr. Secretary Walpole, and has given rise to much discussion in the Legislature. This Bill proposes to incorporate the previous Act of 1848. It gives to Local Boards powers in relation to sewerage, cleansing, and regulation of buildings; and contains many useful clauses tending to simplify sanitary legislation.

Three Medical Reform Bills have been brought forward during the last quarter; and one, prepared and brought in by Mr. Cowper, has passed into committee. Mr. Cowper deserves great credit for the desire he has shown to introduce by law a more catholic feeling amongst the members of the medical profession. For this reason, we would support his measure. The fact is, however, that the only reform required in medicine is self-reformation. There is, in truth, not a single proposition in Mr. Cowper's Bill, either in relation to reciprocity of practice, the promotion of sound medical education, or registration of qualified practitioners, which might not be carried out fully by the profession itself, without any new legislation whatever.

Our cotemporary, the *Medical Times*, suggests the institution of a "Royal Commission of Inquiry." We would suggest the institution of a conference of all the medical, examining, and governing representatives, for the discussion of the whole question of the medical and sanitary requirements of the day. At such a conference, an uniform system of education and examination might be agreed upon, a form of register arranged, —if such an addition to the present *Medical Directory* be required; and a plan adopted for defining the special privileges of different members of the profession. For, after all, the honour of the medical body can only be sustained by the principles of honour present in each individual member; while these will be much more favourably supported by spontaneous action than by any legislative enactment.

*Reports and Returns.*—A "Return", relating to the expenses of the Board of Health, gives the following account:

*Return of Salaries paid or payable to the Officers of the present Board of Health for* 1857.

| | £ | s. | d. |
|---|---|---|---|
| The President (to 24th Sept. 1857, when, under the General Board of Health Continuance Act, 1857, the Vice-President of the Committee of Council on Education was appointed President without salary) | 1,450 | 7 | 4 |
| Private Secretary to President (to 24th September 1857) | 104 | 15 | 7 |
| Medical Officer of the Board | 1,500 | 0 | 0 |
| Secretary | 1,000 | 0 | 0 |
| Assistant Secretary | 600 | 0 | 0 |
| Chief Superintending Inspector | 1,000 | 0 | 0 |
| Two Inspectors (£800 each) | 1,600 | 0 | 0 |
| Surveyor and Draughtsman | 250 | 0 | 0 |
| Four Clerks | 835 | 11 | 11 |
| Office-keeper | 100 | 0 | 0 |
| Three Messengers | 220 | 0 | 0 |
| Housekeeper | 20 | 0 | 0 |
| | £8,680 | 14 | 10 |

The Annual Report of the "National Vaccine Board," shows that the metropolis, during the last year, has been very free from smallpox; and adds the following table, which will interest the statist:

*Deaths from Small-Pox registered in London during Ten Years* 1848-1857 *inclusive.*

| | 1848. | 1849. | 1850. | 1851. | 1852. | 1853. | 1854. | 1855. | 1856. | 1857. |
|---|---|---|---|---|---|---|---|---|---|---|
| 1st quarter | 388 | 228 | 95 | 275 | 389 | 62 | 123 | 323 | 194 | 60 |
| 2nd „ | 381 | 113 | 103 | 209 | 472 | 53 | 122 | 328 | 146 | 27 |
| 3rd „ | 435 | 78 | 109 | 243 | 231 | 42 | 142 | 196 | 108 | 41 |
| 4th „ | 413 | 99 | 191 | 339 | 74 | 60 | 289 | 177 | 74 | 26 |

The report again expresses the importance of securing pure lymph for vaccination, and a careful performance of the operation.

